# Expression of CCR8 and CCX-CKR on Basophils in Chronic Urticaria Is Amplified by IgE-Mediated Activation

**DOI:** 10.3390/biomedicines11061537

**Published:** 2023-05-25

**Authors:** Ewa A. Bartko, Lars H. Blom, Jesper Elberling, Lars K. Poulsen, Bettina M. Jensen

**Affiliations:** Allergy Clinic, Department of Dermatology and Allergy, Copenhagen University Hospital at Gentofte, 2900 Hellerup, Denmark

**Keywords:** basophils, chronic urticaria, skin-homing, CCR8, CCR4, CCX-CKR

## Abstract

Recruitment to the local tissue and alerted phenotype are the hallmarks of basophils in chronic urticaria (CU). Chemokine receptors such as chemokine (C-C motif) receptor 4 (CCR4) or CCR8 have been studied in skin diseases, e.g., atopic dermatitis, but not in CU. In this study, we aimed to define CU’s basophil homing potential and receptor profile and the effect of Omalizumab treatment on these. Unstimulated and activated (anti-IgE, fMLP, C5a, and Substance P) whole blood basophils from 11 Omalizumab-treated CU patients and 10 healthy subjects were investigated with flow cytometry. Unstimulated basophils in CU showed higher expression of the skin-associated (CCR8) and scavenger (CCX-CKR) receptors and lower expression of the lung-associated (CCR3) receptor in contrast to healthy ones. IgE-mediated activation increased the percentage of CCR8 and CCX-CKR in CU compared to healthy group and elevated the expression of the lung-associated chemokine receptor, XCR1, in all groups. A trend of augmented expression of the coagulation cascade (CD87) and fMLP (FPR1) receptors was seen on basophils in CU, while a tendency of reduced expression was seen for itch (IL-31RA) and immunotolerance (CD109) receptors. fMLP and C5a increased the expression of CCR4, CCR8, CCX-CKR, and CD87 and decreased CCR2 and CCR3, though no changes between the groups were found. In conclusion, CU basophils exhibit skin-homing potential amplified by IgE-mediated stimulation.

## 1. Introduction

In chronic urticaria (CU), the number of circulating basophils is lower in the peripheral blood and increased in the lesional skin [1,2]. Moreover, the modified surface expression pattern of CU basophils was described, with increased resting (i.e., unstimulated) expression of high-affinity IgE receptor (FcεRI), substance P receptor (NK1R), CD63, or prostaglandin D2 receptor (CRTH2) [3,4,5,6]. Thus, basophils in CU might have an altered expression profile of receptors involved in the migration (e.g., chemokine receptors) and the skin’s inflammatory response (e.g., coagulation system and immune tolerance-linked receptors).

The expression profile of chemokine receptors in resting basophils of healthy subjects consists of respiratory track-associated receptors, CCR1, CCR2, CCR3, and CXCR2, gut homing receptor, CXCR1, lung/gut/lymphoid tissue/skin-homing marker CXCR4 and gut/lung-associated receptor, CCR5 [7]. Since CU is a skin disease of mild inflammatory background, receptors associated with the cutaneous inflammatory profile and skin homing potential are of interest. The role of CCR4, CCR8, CCR10, and CCR6 or their ligands has been proposed in atopic dermatitis (AD), psoriasis, cutaneous lupus erythematosus, systemic sclerosis, or malignant skin tumors, but not in CU [8,9,10,11]. In addition, the homing potential of cells can be fine-tuned by scavenger receptors, such as CCX-CKR, which blocks the interaction between ligands and their designated receptors and can be found on activated basophils [12,13]. Lastly, it has been shown that approximately 90% of skin-resident lymphocytes are positive for an adhesion-binding antigen, cutaneous lymphocyte-associated antigen (CLA), which has also been reported on basophils from healthy subjects [13].

Basophils in healthy individuals express and respond to activation via CRTH2, FPR1, and CD88. Stimulation with PGD2, fMLP, and C5a can induce the degranulation of basophils and result in their migration. It has been proposed that CU basophils exhibit an altered C5a response in addition to a modified, well-described IgE-FcεRI axis [14,15]. Moreover, since basophils release Th2 cytokines upon IL-31 binding to its heterodimer receptor (IL-31RA/OSMR) [16], an increased concentration of this puritogenic cytokine in CU [17] could be reflected in the expression of the receptor on basophils.

Whether chronic or acute, inflammation suggests disrupted mechanisms of immune tolerance, which are governed by PD-L1 and the IL-10 receptor (CD210). Although the inhibitory mechanisms of PD-L1 signaling and the dampening effect of IL-10 on T cell activation are well known, their role in CU still needs to be addressed. The same applies to CD109, a glycosylphosphatidylinositol-anchored protein that prevents TGF-β mediated dampening effect [18].

In addition to the modified expression of chemokine, activation, and immune tolerance receptors, an altered expression of coagulation system receptors was speculated in CU. Thus, the role of the urokinase system composed of a urokinase-type plasminogen activator (uPA), the uPA receptor—CD87, and two inhibitors have been proposed in chronic urticaria. uPA transforms plasminogen into plasmin, a serine protease that digests extracellular matrix proteins, coagulation system, and basement membrane. Additionally, CD87 can mediate monocyte migration and vitronectin-dependent cell adhesion [19].

In an initial study [13], the expression of a few receptors mentioned above was up-or-down-regulated on basophils from healthy individuals upon anti-IgE stimulation. Therefore, in this study, we sought to broaden the knowledge about the resting expression profile of CU basophils by focusing on chemokine and inflammatory receptors. Additionally, we investigated the effect of IgE and non-IgE mediated stimulations and evaluated an outcome of anti-IgE treatment, Omalizumab (OMZ), on investigated parameters.

## 2. Methods

### 2.1. Study Design

CU patients recruited at Urticaria Clinic, Department of Dermatology, Gentofte Hospital, and healthy subjects were included in this study. Approvals for the project were obtained from the Ethical Committee of the Capital Region of Denmark (H-20034184) and the Danish Data Protection Agency. Patients agreed to participate in the study by signing consent forms. Whole blood samples were drawn from 11 CU patients assigned to the treatment with OMZ—300 mg every 4th week for 12 weeks (3 doses). Blood samples were collected before the 1st injection and after 12 weeks of treatment. Samples from healthy subjects were obtained only once. Three groups of subjects were defined: before OMZ treatment, CU patients—BO, after 12 weeks of OMZ treatment—AO and the healthy subjects’ group—healthy. Due to the basopenic profile, patient no. 3 was excluded from the receptor expression characterization. Additionally, the AO group did not include one CU patient due to changes in the treatment plan. A detailed description of patients can be found in “Elevated, FcεRI-dependent MRGPRX2 expression on basophils in chronic urticaria” [20].

### 2.2. Flow Cytometric Analysis of Basophils

Analysis of surface receptors on basophils was conducted within 4 h after blood sampling into lithium heparin tubes.

Surface staining—In the ratio of 1:1:0.5, whole blood was mixed with a stimulant and one of the six staining antibody mixes. In the same experiment basophils were stimulated either with medium (resting/unstimulated cells), serial dilution of polyclonal goat anti-human IgE (ε) (anti-IgE), 4–4000 ng/mL, (VWR, Radnor, PA, USA), 0.5 µg/mL formyl-methionyl-leucyl-phenylalanine (fMLP) (Sigma-Aldrich, St. Louis, MI, USA ), 1000 ng/mL C5a, (R&D, Minneapolis, MN, USA) or 10 µM Substance P (Sigma-Aldrich, St. Louis, MI, USA) and stained with antibody mixes containing 6 combinations of the following fluorophore labelled antibodies BV711 anti–CCR2 (1D9, BD Franklin Lakes, NJ, USA), BV605 anti–CCR3 (5E8, BD, Franklin Lakes, NJ, USA), PE-Cy™7 anti–CCR4 (1G1, BD, Franklin Lakes, NJ, USA), BV711 anti–CCR6 (11A9, BD, Franklin Lakes, NJ, USA), PE anti–CCR8 (L263G8, Biolegend, San Diego, CA, United States), APC anti–CCR10 (1B5, BD, Franklin Lakes, NJ, USA), PE-Cy™7 anti–CCX-CKR (13E11, Biolegend, San Diego, CA, United States), BV786 anti–CD109 (TEA 2/16, BD, Franklin Lakes, NJ, USA), BV650 anti–CD123 (7G3, BD, Franklin Lakes, New Jersey, USA), BV421 anti–CD210 (3F9, BioLegend, San Diego, CA, United States), FITC anti–CD63 (H5C6, BD, Franklin Lakes, NJ, USA), BV711 anti–CD87 (VIM5, BD, Franklin Lakes, New Jersey, USA), PE anti–CD88 (S5/1, Biolegend, San Diego, CA, United States), BV421 anti–CLA (HECA-452, BD, Franklin Lakes, NJ, USA), PE-CF594 anti–CRTH2 (BM16, BD, Franklin Lakes, NJ, USA), APC anti–FPR1 (W1508bB, Biolegend, San Diego, CA, United States), AF647 anti–IL–31RA (V1-1110, BD, Franklin Lakes, New Jersey, USA), BV421 anti–XCR1 (S15046E, Biolegend, San Diego, CA, United States), PE-Cy™7 anti–PD-L1 (29E.2A3, Biolegend, San Diego, CA, United States). Simultaneous stimulation and staining were conducted for 30 min at 37 °C and followed by 10 min incubation at RT with BD FACS lysing solution (BD, Franklin Lakes, NJ, USA) for fixation and erythrolysis. Subsequently, cells were washed and measured with a flow cytometer, LSR II Fortessa (BD, Franklin Lakes, NJ, USA).

Basophils were defined as either CD123^+^CRTH2^+^ or CCR3^+^CRTH2^+^ single cells (Figure S1). FMO controls for CCR8, CD88, CCX-CKR, FPR1, and PD–L1 were based on basophils stimulated with anti-IgE 1000 ng/mL and were set to 1% (Figure S2A,B). In each of the 6 antibody mixes, resting cells (medium stimulated) were included. For receptors with no FMO, resting cells constituted the baseline for assessing receptors’ expression. Depending on the expression of a receptor on resting basophils, constitutively expressed markers (e.g., CLA) were characterized by the geometric mean of fluorescence—GeoMean, whereas receptors with no resting expression were evaluated as the percentage of receptor-positive basophils—X^+^ basophils. Examples of the receptor expression can be found in Figure S2A,B. Data analysis was carried out with FlowJo software version 10 (TreeStar, Ashland, OR, USA).

Flow values were calculated according to the equation below to address basophils’ stimulation with serial dilution of anti-IgE. A flow value addresses the lowest concentrations of a stimulant, inducing an increase in either the % of receptor-positive basophils or GeoMean, known as sensitivity, but it also covers the maximal response induced by a stimulus. Flow values were only calculated for receptors where anti-IgE stimulation induced a dose-dependent response curve. Higher flow values indicate lower concentrations required to induce changes in the % of receptor-positive basophils.

Flow values = C6^−1^ × Y6 + C5^−1^ × Y5 + C4^−1^ × Y4 + C3^−1^ × Y3 + C2^−1^ × Y2 + C1^−1^ × Y1,

where C6–C1 depicts the anti-IgE concentration in ng/mL (4, 16, 63, 250, 1000, 4000) and Y6–Y1 is either % of receptor-positive basophils or GeoMean value corrected for the background, which is the GeoMean of resting basophils.

### 2.3. Statistical Analysis

Statistical analysis was conducted with GraphPad Prism software, 9.0 (GraphPad Software, San Diego, CA, USA). The significance was determined with an unpaired *t*-test, with a two-sided α-level < 0.05 considered significant; thus, no correction for multiple comparisons was made. The significance values were defined as follows: (*) *p* < 0.05, (**) *p* < 0.01, (***) *p* < 0.001 and (****) *p* < 0.0001. Values in parenthesis show mean with SD.

## 3. Results 

### 3.1. Receptor Expression on Resting Basophils—Increased Expression of CCR8 and CCX-CKR and Decreased Expression of CCR3 on Unstimulated (Resting) Basophils in CU

To investigate the potential effect of OMZ treatment on the surface receptors, we first evaluated their expression on resting basophils. Two groups of receptors were studied: chemokine and miscellaneous (Table 1).

**Figure 1:**
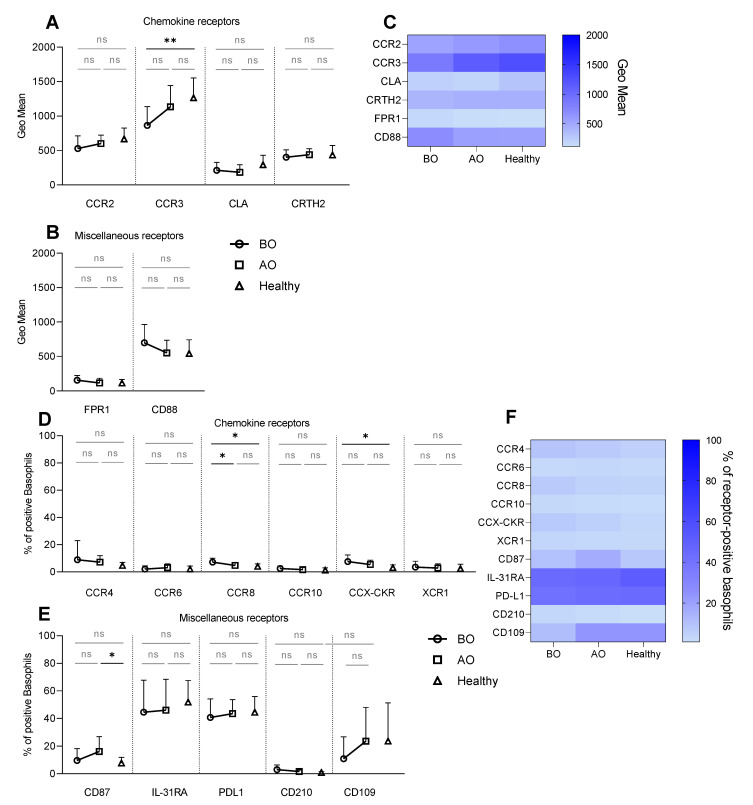
Resting expression—chemokine and miscellaneous receptors. Surface expression of chemokine and miscellaneous receptors on resting basophils. (**A**–**C**) Receptor expression shown as GeoMean, (**D**–**F**) Receptor expression shown as a percentage of receptor-positive basophils. (**A**) GeoMean of CCR2, CCR3, CLA, and CRTH2. (**B**) GeoMean of FPR1 and CD88. (**C**) Heat map summarizing resting expression of investigated receptors measured as GeoMean. (**D**) Percentage of basophils positive for CCR4, CCR6, CCR8, CCR10, CCX-CKR, and XCR1. (**E**) Percentage of CD87^+^, IL-31RA^+^, PD-L1^+^, CD210^+^, and CD109^+^ basophils. (**F**) Heat map summarizing resting expression of investigated receptors measured as a percentage of receptor-positive basophils. BO and AO refer to patients’ groups before and after Omalizumab treatment, respectively; dots, squares, and triangles refer to BO (n = 10), AO (n = 9), and healthy (n = 10) groups, respectively; % of CCR8^+^ basophils in BO group is presented for nine subjects due to staining issues; applied statistics—unpaired *t*-test; ns—non-significant, *p* > 0.05, *—*p* ≤ 0.05, **—*p* ≤ 0.01; Mean ± SD; SD—standard deviation.

A selected panel of chemokine receptors (Table 1) was analyzed on basophils from healthy and patients with CU to define the tissue homing potential. Basophils from all groups constitutively expressed CCR2, CCR3, CLA, and CRTH2 (Figure 1A,C). Expression of CCR3 was lower (GeoMean: 864 ± 27 vs. 1134 ± 308) in the BO group compared to healthy subjects, whereas the expression of CRTH2 and CLA was similar between the groups (Figure 1A,C). The percentage of resting basophils positive for CCR4, CCR8, and CCX-CKR was below 10% in all groups (Figure 1D,F). However, the expression of CCR8 (7.2% ± 2.9 vs. 4% ± 1.8) and CCX-CKR (7.5% ± 4.8 vs. 3.1% ± 2) was higher on basophils in CU subjects before treatment compared to healthy (Figure 1D,F). Furthermore, OMZ treatment decreased the expression of CCR8 (4.7% ± 1.9) (Figure 1D,F).

The receptor expression on resting basophils was further explored by investigating tolerance-linked receptors—CD210, PD-L1, CD109; itch receptor—IL-31RA; the urokinase-type plasminogen activator receptor—CD87; fMLP receptor—FPR1 and C5a receptor—CD88 (Table 1). Approximately 50% of resting basophils were positive for either IL-31RA or PD-L1 in all groups (Figure 1E,F). Healthy and BO individuals showed a similar percentage of CD87^+^ basophils, which was increased with the treatment (16.1% ± 10.8) in contrast to healthy (7.8% ± 4.1) (Figure 1E,F). Comparable resting expressions of CD109 (Figure 1E,F), FPR1, and CD88 were found in all groups (Figure 1B,C).

### 3.2. IgE-Mediated Stimulation Elevates the Expression of CCR8 and CCX-CKR, Boosting the Chemokine Response Potential of Basophils in CU

As IgE-dependent activation of CU basophils shows higher sensitivity, being responsive to suboptimal stimulant concentrations, we decided to explore if there was an effect of IgE-mediated stimulation on the expression of chemokine and miscellaneous receptors [20] on basophils from healthy and CU patients before and after OMZ treatment. Basophils were stimulated with serial dilution of anti-IgE (4–4000 ng/mL). The dose-dependent response to the stimulation was summarized in one value named a flow value.

**Figure 2:**
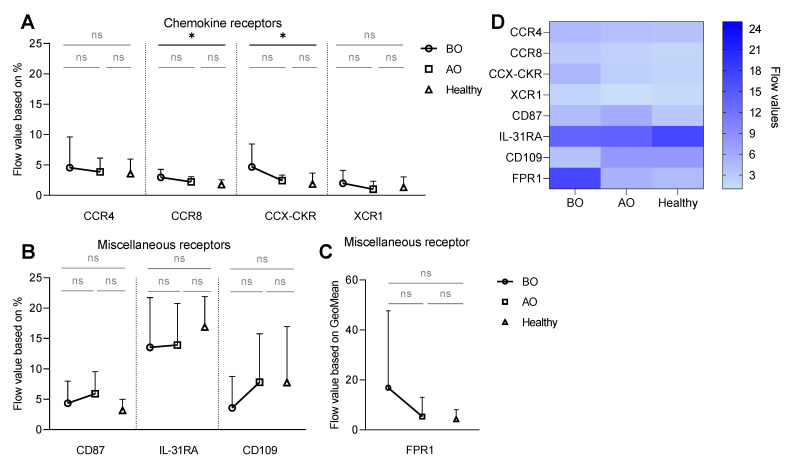
IgE-mediated expression of chemokine and miscellaneous receptors is altered on CU basophils. Whole blood basophils were stimulated with serial dilution of anti-IgE (4–4000 ng/mL). Surface expression of chemokine and miscellaneous receptors was defined as flow values, either based on the percentage of receptor-positive basophils or GeoMean. (**A**) Chemokine receptors—flow values based on the % of CCR4^+^, CCR8^+^, CCC-CKR^+^, and XCR1^+^ basophils. (**B**) Miscellaneous receptors—flow values based on the % of CD87^+^, IL-31RA^+^_,_ and CD109^+^ basophils. (**C**) FPR1 flow value based on the GeoMean. (**D**) Heat map summarizing flow values of investigated receptors. BO and AO refer to patients’ groups before and after Omalizumab treatment, respectively; dots, squares, and triangles represent BO (n = 10), AO (n = 9), and healthy (n = 10) groups, respectively; % of CCR8^+^ basophils in BO group is presented for nine subjects due to staining issues, applied statistics—unpaired *t*-test; ns—non-significant, *p* > 0.05, *—*p* ≤ 0.05; Mean ± SD; SD—standard deviation.

The anti-IgE stimulation increased the frequency of CCR4, CCR8, XCR1, and CCX-CKR positive basophils in a dose-dependent manner in all groups (Figure S3A). Interestingly, the BO CU group showed higher expression of CCX-CKR (flow value: 4.7 ± 3.8) and CCR8 (1.9 ± 1.9) in comparison to healthy subjects (CCX-CKR–1.9 ± 1.9; CCR8–1.8 ± 0.8) (Figure 2A,D).

Within the group of miscellaneous receptors, anti-IgE stimulation induced a dose-dependent increase in the percentage of CD87^+^ basophils and, interestingly, a decrease in the frequency of IL-31RA^+^ in all groups (Figure S4). The BO group showed the lowest expression of CD109 (flow value: BO: 3.6 ± 5.2 vs. AO: 7.8 ± 8 vs. Healthy: 7.8 ± 9.2) (Figure 2B,D). Interestingly, the increased expression of FPR1 (Figure S4) tended to be reduced by OMZ treatment, resembling values of healthy subjects (Figure 2C,D).

### 3.3. Non-IgE-Mediated Stimulation Mimics the Effect of the IgE-FcεRI Axis

Since CU basophils have been described for their altered non-IgE induced activation, e.g., C5a, we investigated the effect of G protein-coupled receptors ligands: fMLP, C5a, and SP on the expression of chemokine and miscellaneous receptors.

**Figure 3:**
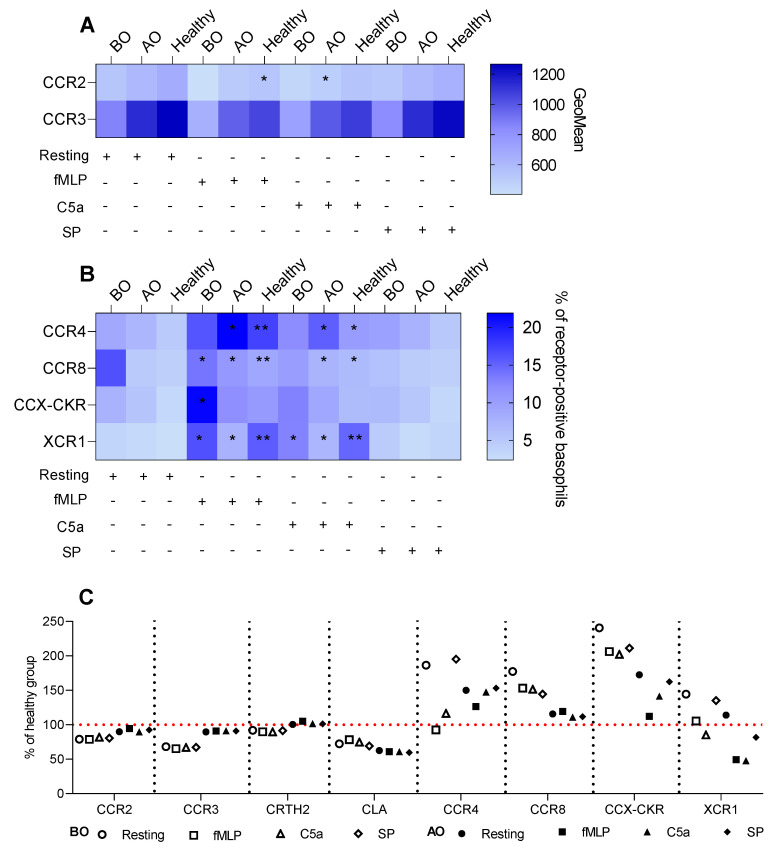
Non–IgE induced changes in chemokine receptors expression. Whole blood basophils were activated with either fMLP, C5a, or SP. Surface expression of chemokine receptors was depicted as a percentage of receptor-positive basophils or GeoMean. (**A**) Heat map summarizing expression of CCR2 and CCR3 presented as GeoMean—resting vs. stimulated basophils. (**B**) Heat map summarizing the percentage of CCR4, CCR8, CCX-CKR, and XCR1 positive basophils—resting vs. stimulated basophils. (**C**) % of the healthy group for BO and AO chemokine receptors; the red line indicates the healthy group as a reference. BO and AO refer to patients’ groups before and after Omalizumab treatment, respectively; BO (n = 10), AO (n = 9), and healthy (n = 10) groups; % of CCR8^+^ basophils in the BO group is presented for nine subjects due to staining issues; applied statistics—unpaired *t*-test, *p* > 0.05, *—*p* ≤ 0.05, **—*p* ≤ 0.01; Mean ± SD.

Compared to resting expression, C5a, and fMLP stimulations tended to reduce expressions of CCR2 and CCR3 in all groups. Though, only C5a in the AO group (GeoMean: 600 ± 122 vs. 485 ± 100) and fMLP in the healthy group (669 ± 157 vs. 534 ± 127) significantly decreased the expression of CCR2 (Figure 3A). fMLP and C5a elevated the CCR4 expression on resting basophils in healthy (rest.: 4.7% ± 2.2 vs. fMLP: 17.3% ± 11.6 vs. C5a: 10.4% ± 6.5) and AO (7.1% ± 4.8 vs. 21.9% ± 15.9 vs. 15.3% ± 9.3) groups (Figure 3B). Additionally, fMLP and C5a increased the expression of CCR8 (4% ± 1.8 vs. 9% ± 5 vs. 6.7% ± 3.3) and XCR1 (2.4% ± 3.2 vs. 15.5% ± 13.7 vs. 15% ± 12) on basophils in the healthy group. The OMZ-treated group also showed an increase of CCR8^+^ (4.8% ± 1.9 vs. 10.7% ± 6.2 vs. 7.5% ± 2.6) and XCR1^+^ (2.8% ± 3.2 vs. 7.6% ± 6 vs. 7.1% ± 4.7) basophils (Figure 3B). Interestingly, fMLP elevated the expression of CCR8 (7.2% ± 2.9 vs. 13.8% ± 6.2) and XCR1 (3.9% ± 4.3 vs. 16.3% ± 15.7) on basophils in the BO group; however, the C5a stimulation increased the frequency of XCR1^+^ (12.8% ± 12.4) but not CCR8^+^ cells (Figure 3B). Only the percentage of resting CCX-CKR^+^ basophils in the BO group was increased by fMLP (7.5% ± 4.8 vs. 21.6% ± 19.4) (Figure 3B).

Interestingly, SP failed to trigger any changes in all groups (Figure S5A–C). The differences observed in the expression of CCR2, CCR3, and the percentage of CCX-CKR^+^ basophils between the groups were driven by the groups’ effect (baseline expression is different between the groups) and not by a stimulant (Figure S5D,E).

**Figure 4:**
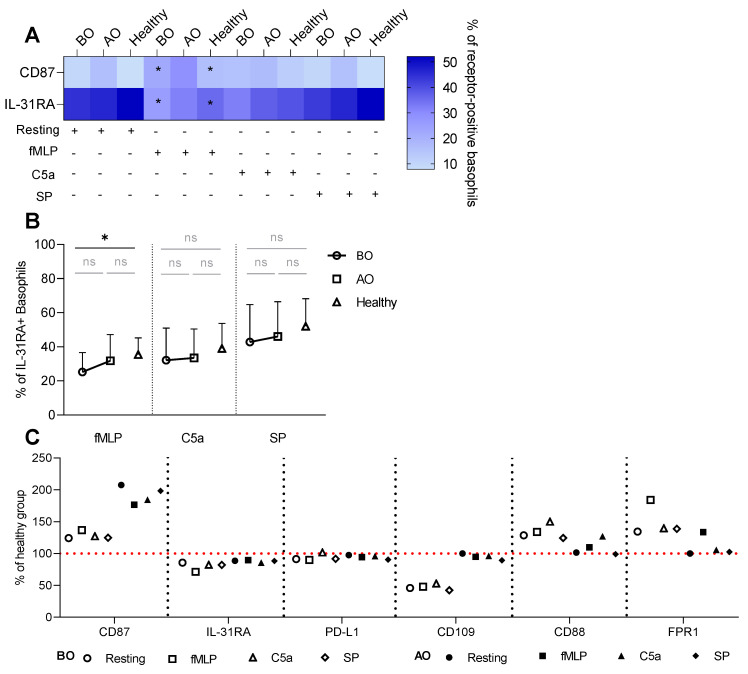
Non-IgE induced changes in the miscellaneous receptors’ expression. Whole blood basophils were activated with either fMLP, C5a, or SP. Surface expression of miscellaneous receptors was defined as % of receptor-positive basophils. (**A**) Heat map summarizing the percentage of CD87 and IL-31RA positive basophils—resting vs. stimulated basophils. (**B**) % of IL-31RA^+^ basophils—BO vs. AO vs. Healthy. (**C**) % of the healthy group for BO and AO miscellaneous receptors, the red line indicates the healthy group as reference. BO and AO refer to patients’ groups before and after Omalizumab treatment, respectively; BO (n = 10), AO (n = 9), and healthy (n = 10) groups; applied statistics—unpaired *t*-test; ns—non-significant, *p* > 0.05, *—*p* ≤ 0.05; Mean ± SD; SD—standard deviation.

Similar to the IgE-dependent stimulation, fMLP augmented the expression of resting CD87^+^ basophils in BO (rest: 9.7% ± 8.5 vs. fMLP: 22.3% ± 15) and healthy (7.8% ± 4.1 vs. 16.3% ± 8.8) groups (Figure 4A). Additionally, fMLP reduced the expression of IL-31RA compared to resting basophils in BO (44.5% ± 23.3 vs. 25.2% ± 11.4) and healthy (51.9% ± 15.7 vs. 35.4% ± 9.8) groups (Figure 4A).

By comparing groups within each stimulus, we detected a lower fMLP-induced expression of IL-31RA in the BO group (25.2% ± 11.4) in contrast to the healthy (35.4% ± 9.8) (Figure 4B). SP stimulation was generally ineffective in changing the expression of all miscellaneous receptors (Figure 4). The difference in the % of CD87^+^ basophils between the groups resulted from the groups’ effect (Figure S6F). The decrease in the expression of CD88 and FPR1 while stimulated with their ligands was most likely mediated by the competition between the ligand and the staining antibody for the binding site on the receptor or by receptor internalization upon binding of a ligand (Figure S6D,E).

In general, OMZ treatment tended to shift the receptor expression pattern toward the healthy group (Figure 3C and Figure 4C). However, there were three exceptions—two chemokine receptors: CLA, XCR1, and CD87 that were decreased or increased in comparison to healthy, respectively (Figure 3C and Figure 4C).

## 4. Discussion

In healthy individuals, basophils are circulating white blood cells that can potentially migrate to tissues while called upon [2,21,22]. The observation of a reduced basophil blood count and increased number of basophils in biopsies from lesional skin suggests infiltration of these cells to the affected tissue in CU patients [23,24]. As healthy resting basophils express CCR1, CCR2, CCR3, CCR5, CXCR1, CXCR2, and CXCR4 [7,25], we speculated that CU basophils might present a skin-skewed migratory profile that can be encapsulated by expression of CCR4, CCR8, CCR6, CCR10, and CLA receptors. Studies on T cells showed that CLA^+^ lymphocytes known for their cutaneous recruitment co-expressed CCR4 and CCR10, indicating their involvement in skin retention [8,11]. Moreover, ligands for CCR4—CCL17 and CCL22, and CCR10—CCL27 have been associated with AD, psoriasis, cutaneous lymphomas, and systemic sclerosis [26,27]. In addition, CCR8 and CCR6 were described for their role in atopic skin diseases [10] and psoriasis [9], respectively. Indeed, we observed a higher frequency of CCR8^+^ resting basophils and the same tendency for CCR4 in CU subjects compared to healthy controls. In contrast, the lack of CCR6 and CCR10 expression suggests a less skin inflammatory profile of CU than AD and psoriasis, where both receptors are well-described for their role. To further support the cutaneous homing potential of CU basophils, we noted a reduced expression of the lung-associated homing receptors CCR3 [28] and CCR2 and no expression of yet another pulmonary marker, XCR1. In addition, the chemokine response potential was strengthened by the elevated expression of CCX-CKR, a scavenger receptor, with binding properties towards the ligands for the lymph node receptor, CCR7 (CCL19, CCL21) [29], and for the gut receptor, CCR9 (CCL25) [30]. Increased expression of CCX-CKR implies amplified control over chemokine bioavailability that prevents the interaction of ligands with their cognate receptors, thereby diminishing the possibility of basophils to home to either lymph nodes or the gut. Surprisingly, resting CU basophils displayed a tendency of reduced CLA and no changes in CRTH2 expression contrary to healthy. The latter finding contradicts the study by Oliver et al., where a decreased expression of CRTH2 was defined on CU basophils [6]. To broaden our understanding of basophil recruitment, we investigated the effect of IgE and non-IgE mediated (fMLP, C5a, and SP) stimulations. Within the latter group, SP was unsuccessful in eliciting receptor expression changes. Moreover, neither of the stimulants altered CCR6, CCR10, and CRTH2 expressions illustrating no involvement of these receptors in basophils homing in CU. Although the IgE-dependent increase in the percentage of CCR4^+^, CCR8^+^, and CCX-CKR^+^ basophils was observed in all groups, the sensitivity of CU basophils compared to healthy was elevated only for CCR8 and CCX-CKR, with a similar tendency for CCR4. This suggests that the threshold of the IgE-mediated stimulation for CU basophils is substantially lower to potentiate their resting skin-like homing profile. Interestingly, we observed a similar profile for fMLP and C5a stimulations. In addition, the diminished CCR2 and CCR3 expressions on CU basophils were maintained after IgE and non-IgE mediated activation limiting the movement of cells to respiratory tract tissues. Surprisingly, both stimulation pathways increased the count of XCR1^+^ basophils in all groups implying that XCR1 expression on basophils is independent of the route of activation and disease status. Overall, the cutaneous homing potential of resting CU basophils is amplified by the activation through the IgE-FcεRI axis and, to a lesser degree, by the fMLP and C5a stimulations.

Supplementary to chemokine receptor expression, we investigated the expression of receptors/markers linked to immune tolerance, histamine-independent itch, and non-IgE mediated stimulation. Out of three immune tolerance-linked receptors, CD210 expression was not detected. In contrast, the disease-independent expression of PD-L1 was measured in all groups, signifying basophils’ ability to regulate immune responses. Though not significantly, the expression of inflammatory promoting [31] receptor, CD109 that is induced by IL-4 on Th2 cells [32], was reduced in CU patients. As inflammatory properties of CD109 have been proven in the context of the systemic autoimmune chronic disease, rheumatoid arthritis, the mechanism of chronic events in urticaria might be of a less severe, fibroblast-independent nature. In this study, we also observed a tendency of a lower percentage of IL-31RA^+^ circulating CU basophils, which contradicts an increased CU serum level [33] and intracellular IL-31 in basophils in CSU lesions [16]. This opposite trend could be rationalized by IL-31-mediated secretion of IL-4 and IL-13, which in local tissues would induce a more favorable effect than in circulation. Additionally, we observed a higher expression of FPR1 than in healthy subjects suggesting that non-IgE-dependent stimulation of CU basophils could provide a more robust response than in healthy subjects. Interestingly, healthy and urticaria basophils showed expression of CD87, urokinase-type plasminogen activator receptor—uPAR. This receptor’s exact role in basophils has not been investigated; however, it is suggested to play a role in epithelial wound repair and tissue remodeling in asthma [34] as well as in cell migration and adhesion. IgE and non-IgE mediated stimulations did not change the relation of CD87 and IL-31RA expression between healthy and CU basophils; however, it augmented and decreased the percentage of both receptors, respectively. While the changes seen in the expression of CD87 resembled a pattern determined for chemokine receptors associating this marker with the migration of basophils, the dynamic of IL-31RA shows that stimulation of basophils with degranulation-inducing secretagogues reduces their capacity to IL-31 mediated cytokine release. Additionally, we detected no changes in the expression of CD210, PD-L1, and CD88, whereas FPR1 was increased by both stimulants, with CU basophils presenting a tendency of higher expression that could indicate a more reactive phenotype, fitting into chemokine receptors’ profile.

As a part of the study, we also evaluated the effect of the treatment with an anti-IgE monoclonal antibody, OMZ. Overall, OMZ modified the expression of most of the receptors on resting CU basophils, elucidating the connection between the IgE-mediated activation and investigated markers. As the IgE-mediated stimulation potentiated the receptor profile of resting CU basophils, it is reasonable to suspect a regulating effect of anti-IgE treatment on receptor expression. However, it was intriguing to see a comparable effect of OMZ on non-IgE-mediated stimulation. This could indicate a connection between both pathways regulated by the IgE’s presence. In addition, OMZ increased the expression of CD87 and did not affect the IL-31RA, showing that the expression of some receptors did not resemble healthy subjects’ profiles after the treatment. The expression of these receptors could be utilized as markers of OMZ efficacy; however, additional studies with a larger group of participants should be conducted to support the rationale of this assumption.

In this study, we presented for the first time a fingerprint of chemokine receptors that may guide the skin-homing profile of CU basophils (Table S1). As the present study was conducted on a limited number of patients, trends observed for some receptors lack statistical power. A larger group of participants is required to validate and further investigate the relevance of these receptors and possibly seek patterns within patients responding and non-responding to the OMZ treatment. Additionally, as this study was focused on the expression of receptors, their functionality, i.e., binding of a ligand or ligands to their cognate receptors and intracellular events following the binding, should be studied. Moreover, the intracellular receptor expression and localization could help to address a plausible association of chemokine and degranulation receptors. Lastly, investigating the RNA levels of receptors could help better understand the dynamic of receptors in basophils from both healthy and chronic urticaria individuals. 

## Figures and Tables

**Table 1:** Investigated chemokine and miscellaneous receptors. The table depicts the 17 investigated chemokine and miscellaneous receptors. Each group was divided into receptors whose expression was shown as a percentage of receptor-positive basophils (%) and the geometric mean of fluorescence (GeoMean).

	%	Geo Mean
***Chemokine receptors***	CCR4	CCR2
	CCR6	CCR3
	CCR8	CLA
	CCR10	CRTH2
	CCX-CKR	
	XCR1	
***Miscellaneous receptors***	CD87	FPR1
	IL-31RA	CD88
	PD-L1	
	CD210	
	CD109	

## Data Availability

Data available on request due to restrictions.

## Supplementary Materials

The following supporting information can be downloaded at: https://www.mdpi.com/article/10.3390/biomedicines11061537/s1. Figure S1: Gating strategy. Figure S2: Expression of investigated receptors on basophils in CU and healthy subjects–representative dot plots. Figure S3: A IgE mediated dose-dependent increase in the expression of CCR4, CCR8, CCX–CKR, and XCR1. Figure S4: IgE mediated stimulation—an expression of CD87, IL-31RA, PD-L1, CD109, FPR1, CD88 and CD210. Figure S5: Non–IgE induced activation—the expression of chemokine receptors. Figure S6: Non-IgE mediated activation of basophils—the expression of miscellaneous receptors. Figure S7: B IgE-mediated stimulation—an expression of CCR2, CCR3, CLA, CRTH2, CCR6, and CCR10. Table S1: Overview of receptor expression on basophils from chronic urticaria patients and healthy subjects.

## Abbreviations

AD: Atopic dermatitis, AO: After Omalizumab treatment, BO: Before Omalizumab treatment, C5a: Complement component 5a, CCL: Chemokine, CCR: chemokine receptor, CCX-CKR: Atypical chemokine receptor 4, CD: Cluster of differentiation, CLA: Cutaneous lymphocyte-associated antigen, CRTH2: Prostaglandin D2 receptor 2, CU: Chronic urticaria, CXCR: C-X-C chemokine receptor, FcεRI: high-affinity IgE receptor, fMLP: formyl-methionyl-leucyl-phenylalanine, FMO: Fluorescence minus one, FPR1: formyl-methionyl-leucyl-phenylalanine receptor, GeoMean: Geometric Mean, IL- Interleukin, IL-31RA: Interleukin-31 receptor subunit alpha, OMZ: Omalizumab, OSMR: Oncostatin-M-specific receptor subunit beta, PD-L1: Programmed cell death ligand 1, PGD2: Prostaglandin D2, SP: Substance P, TGF-β: Transforming growth factor β, uPA: urokinase-type plasminogen activator, uPAR: Urokinase plasminogen activator surface receptor, RT: Room temperature, XCR1: Chemokine XC receptor 1.
