# Supplementary material for: Expression of CCR8 and CCX-CKR on Basophils in Chronic Urticaria Is Amplified by IgE-Mediated Activation
Source: Biomedicines. 2023 May 25;11(6):1537. doi: 10.3390/biomedicines11061537 (PMC10295731; doi:10.3390/biomedicines11061537)
Supplement: Supplementary file 1 [file biomedicines-11-01537-s001.zip › biomedicines-1924352-supplementary.pdf]

Supplementary figures

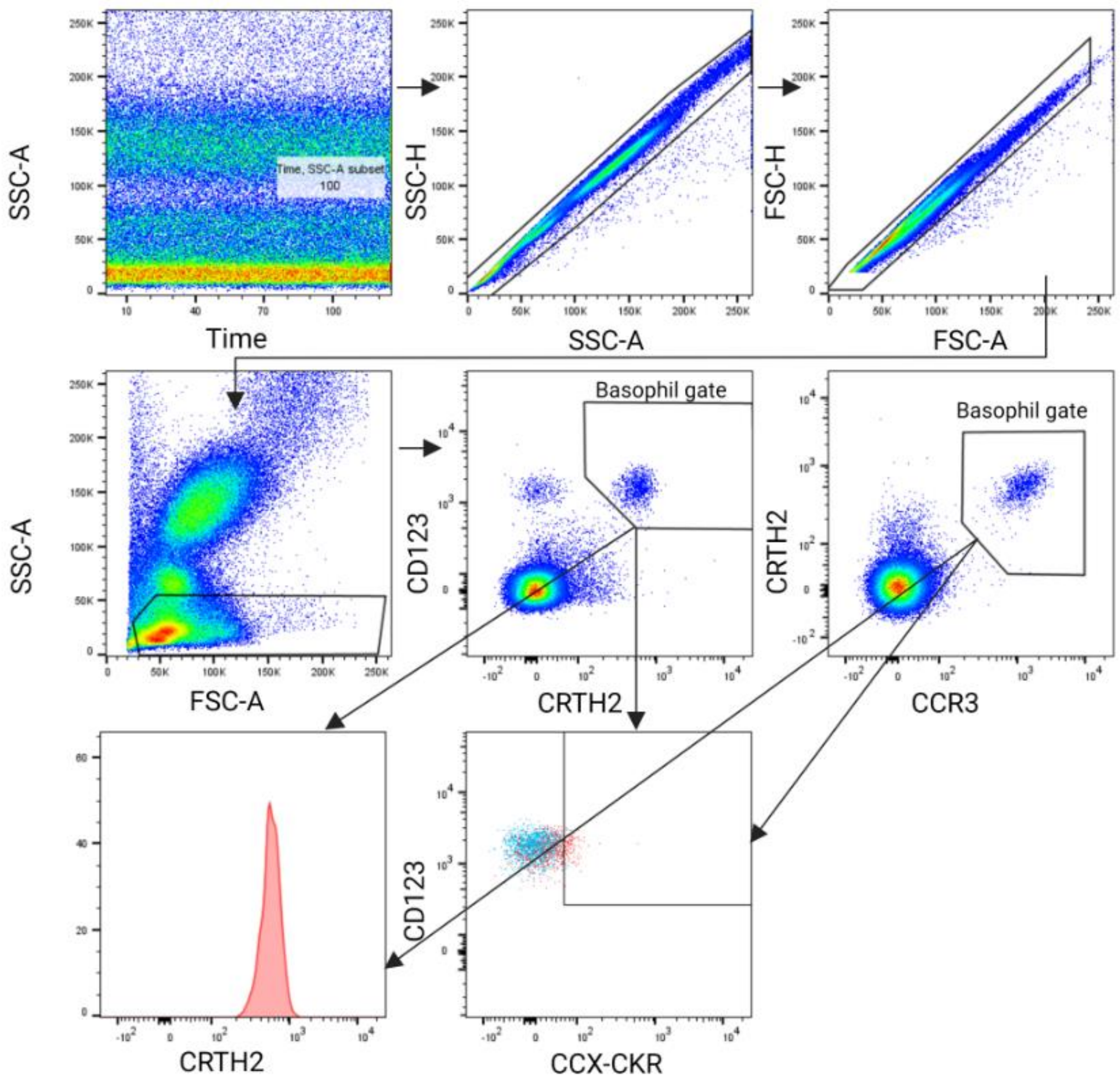

Figure S1 Gating strategy

The following gating approach was applied for the identification of basophils: SSC-A vs time, SSC-A vs SSC-H, FSC-A vs FSC-H, FSC-A vs SSC-A, and CRTH2 vs CD123/ CCR3 vs CRTH2 (basophil gate). Expression of receptors was investigated as GeoMean (histogram bottom panel, left column, e.g., CRTH2) or % of marker^{+} basophils (e.g., red dot plot, bottom panel, middle column). FMO controls were used for several receptors, e.g., CCX-CKR (blue dot plot, bottom panel, middle column).

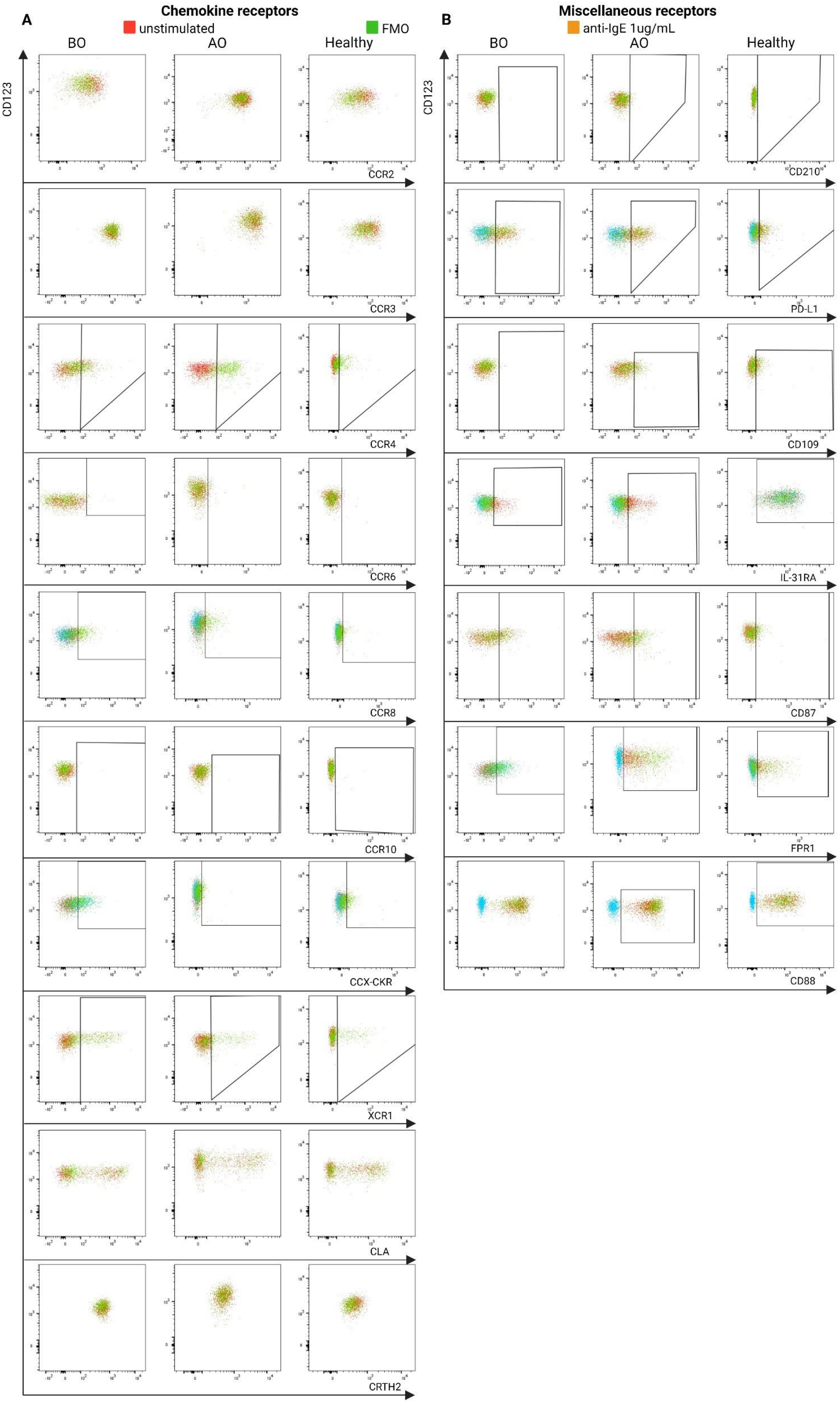

A
Chemokine receptors
unstimulated
FMO
BO
AO
Healthy
CD123
CCR2
CCR3
CCR4
CCR6
CCR8
CCR10
CCX-CKR
XCR1
CLA
CRTH2
B
Miscellaneous receptors
anti-IgE 1ug/mL
BO
AO
Healthy
CD123
CD210
PD-L1
CD109
IL-31RA
CD87
FPR1
CD88

Figure S2 Expression of investigated receptors on basophils in CU and healthy subjects – representative dot plots Dots plots show the expression of chemokine (A) and miscellaneous (B) receptors on basophils from CU patients before (BO) and after (AO) Omalizumab treatment and from healthy subjects. The overlayed dots plots depict the expression of receptors on unstimulated (red dots), FMO control (blue dots), and anti-IgE 1000 ng/mL stimulated (green dots) basophils. Gates consist of receptor-positive cells.

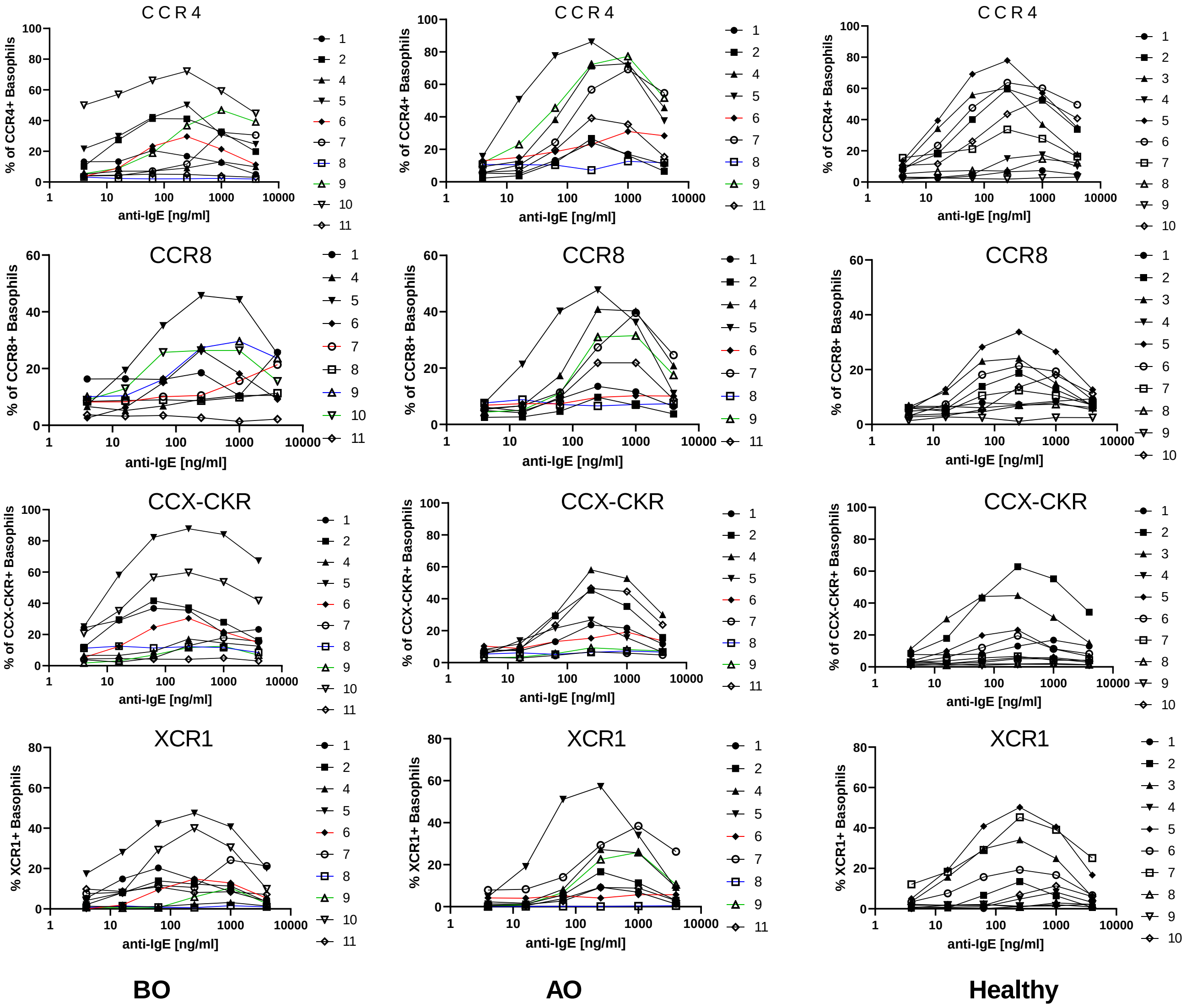

Figure S3, A IgE mediated dose-dependent increase in the expression of CCR4, CCR8, CCX–CKR, and XCR1 Whole blood basophils were stimulated with serial dilution of anti-IgE (4-4000 ng/mL) – X-axis. Change in the characterized receptors is presented on the Y-axis as % of receptor-positive basophils.

BO and AO refer to patients' groups before and after Omalizumab treatment, respectively; Left, middle, and right panels refer to BO (1-2, 4-11), AO (1-2, 4-9, 11), and healthy (1-10) groups, respectively; % of $CCR8^+$ basophils in the BO group is presented for 9 subjects due to staining issues.

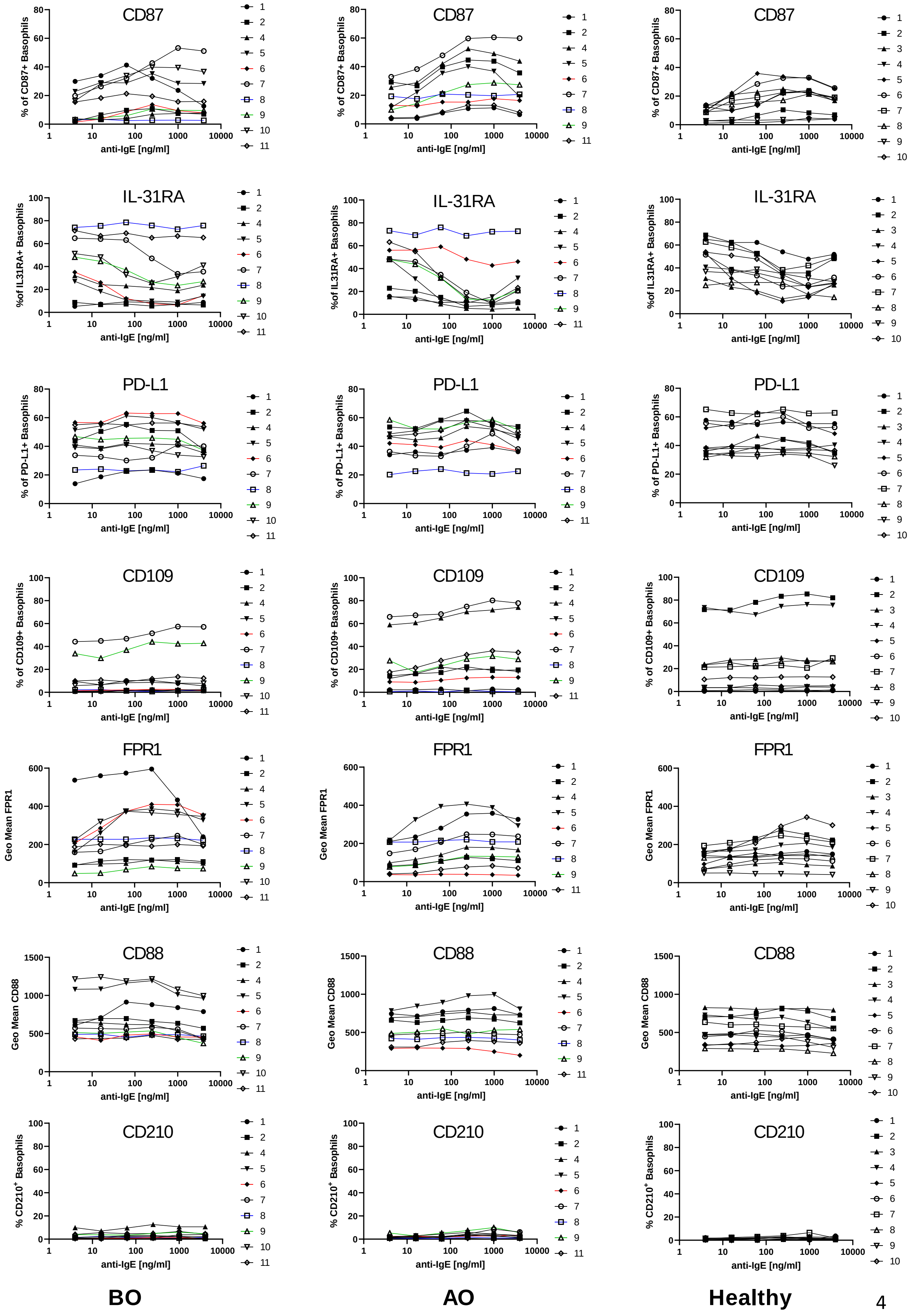
CD87
% of CD87+ Basophils
anti-IgE [ng/ml]
IL-31RA
% of IL31RA+ Basophils
%of IL31RA+ Basophils
PD-L1
% of PD-L1+ Basophils
CD109
% of CD109+ Basophils
FPR1
Geo Mean FPR1
CD88
Geo Mean CD88
CD210
% CD210+ Basophils
BO
AO
Healthy

Figure S4 IgE mediated stimulation – an expression of CD87, IL-31RA, PD-L1, CD109, FPR1, CD88 and CD210

Whole blood basophils were stimulated with serial dilution of anti-IgE (4-4000 ng/mL) – X-axis. Change in the characterized receptors is presented on the Y-axis as either % of receptor-positive basophils or GeoMean.

BO and AO refer to patients' groups before and after Omalizumab treatment, respectively; Left, middle, and right panels refer to BO (1-2, 4-11), AO (1-2, 4-9, 11), and healthy (1-10) groups, respectively

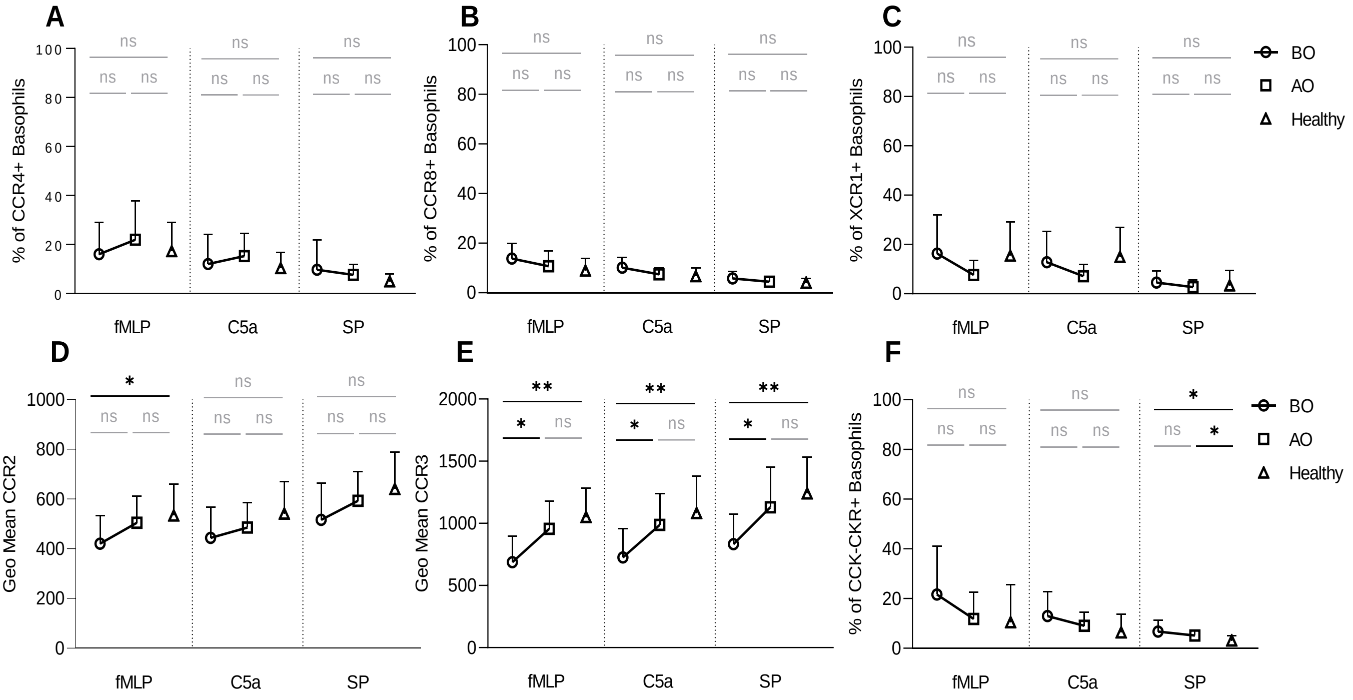

Figure S5 Non–IgE induced activation – the expression of chemokine receptors

Whole blood basophils were stimulated with either fMLP, C5a, or SP. Surface expression of chemokine receptors was depicted as % of receptor-positive basophils or GeoMean. **A–C.** % of $CCR4^+$, $CCR8^+$, $XCR1^+$ basophils – BO vs. AO vs. healthy **D and E**. GeoMean of CCR2 and CCR3 – BO vs. AO vs. healthy **F.** % of CCX-CKR^+ basophils – BO vs. AO vs. Healthy

BO and AO refer to patients' groups before and after Omalizumab treatment, respectively; dots, squares, and triangles represent BO (1-2, 4-11), AO (1-2, 4-9, 11), and healthy (1-10) groups, respectively; % of $CCR8^+$ basophils in BO group is presented for 9 subjects due to staining issues; applied statistics – unpaired t-test; ns – non-significant, $p > 0.05$, * - $p \leq 0.05$, ** - $p \leq 0.01$; Mean ± SD; SD – standard deviation

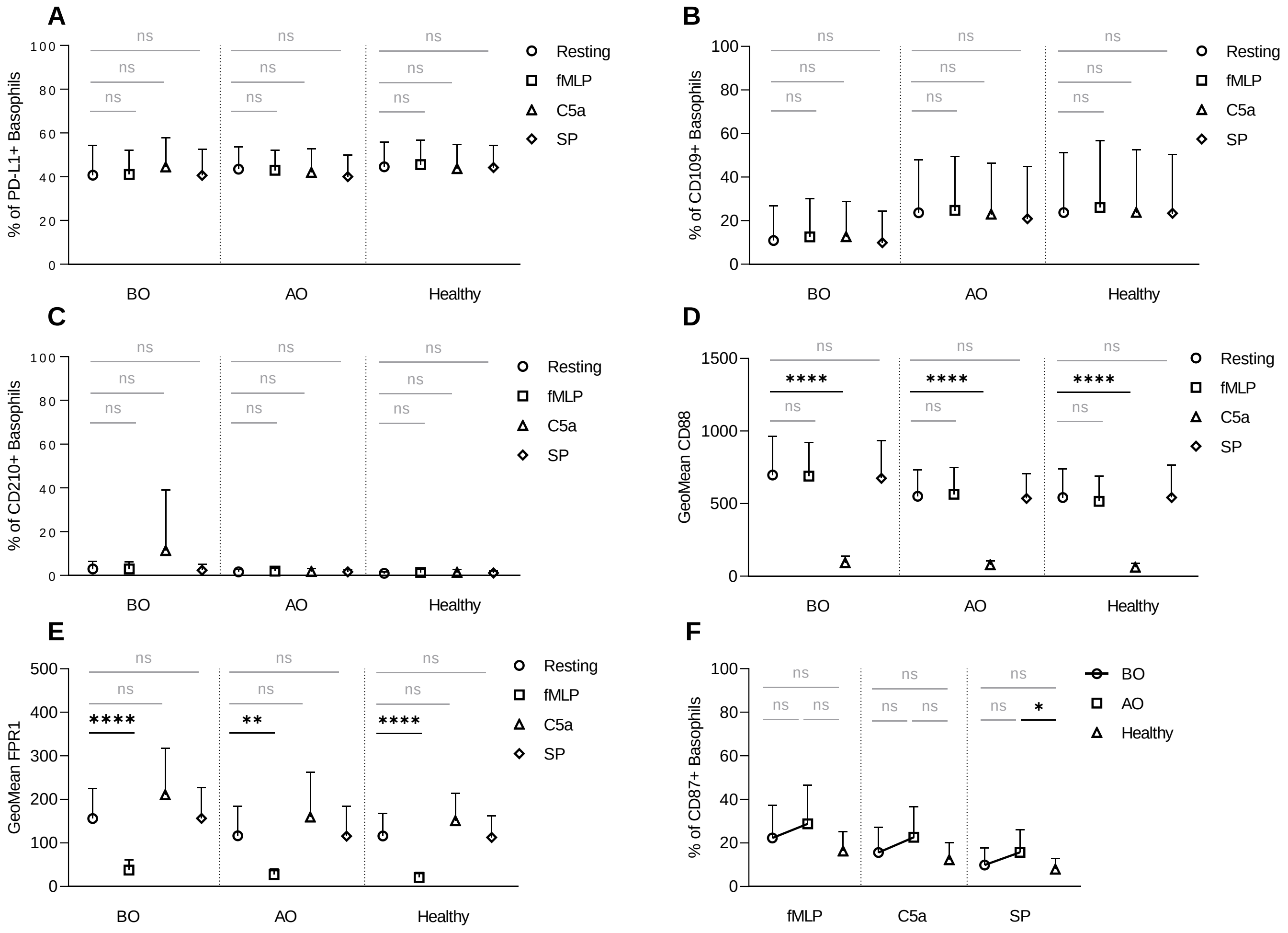

Figure S6 Non-IgE mediated activation of basophils - the expression of miscellaneous receptors

Whole blood basophils were stimulated with either fMLP, C5a, or SP. Surface expression of miscellaneous receptors was defined as % of receptor-positive basophils or GeoMean. **A-C.** % of PD-L1, CD109, and $CD210^{+}$ basophils – resting vs. stimulated basophils **D and E.** GeoMean of CD88 and FPR1 - resting vs. stimulated basophils **F.** % of CD87 $^{+}$ basophils - BO vs. AO vs. Healthy

BO and AO refer to patients' groups before and after Omalizumab treatment, respectively; BO (1-2, 4-11), AO (1-2, 4-9, 11), and healthy (1-10) groups; applied statistics – unpaired t-test; ns – non-significant, $p > 0.05$, * - $p \leq 0.05$, ** - $p \leq 0.01$, **** - $p \leq 0.0001$; Mean ± SD; SD – standard deviation

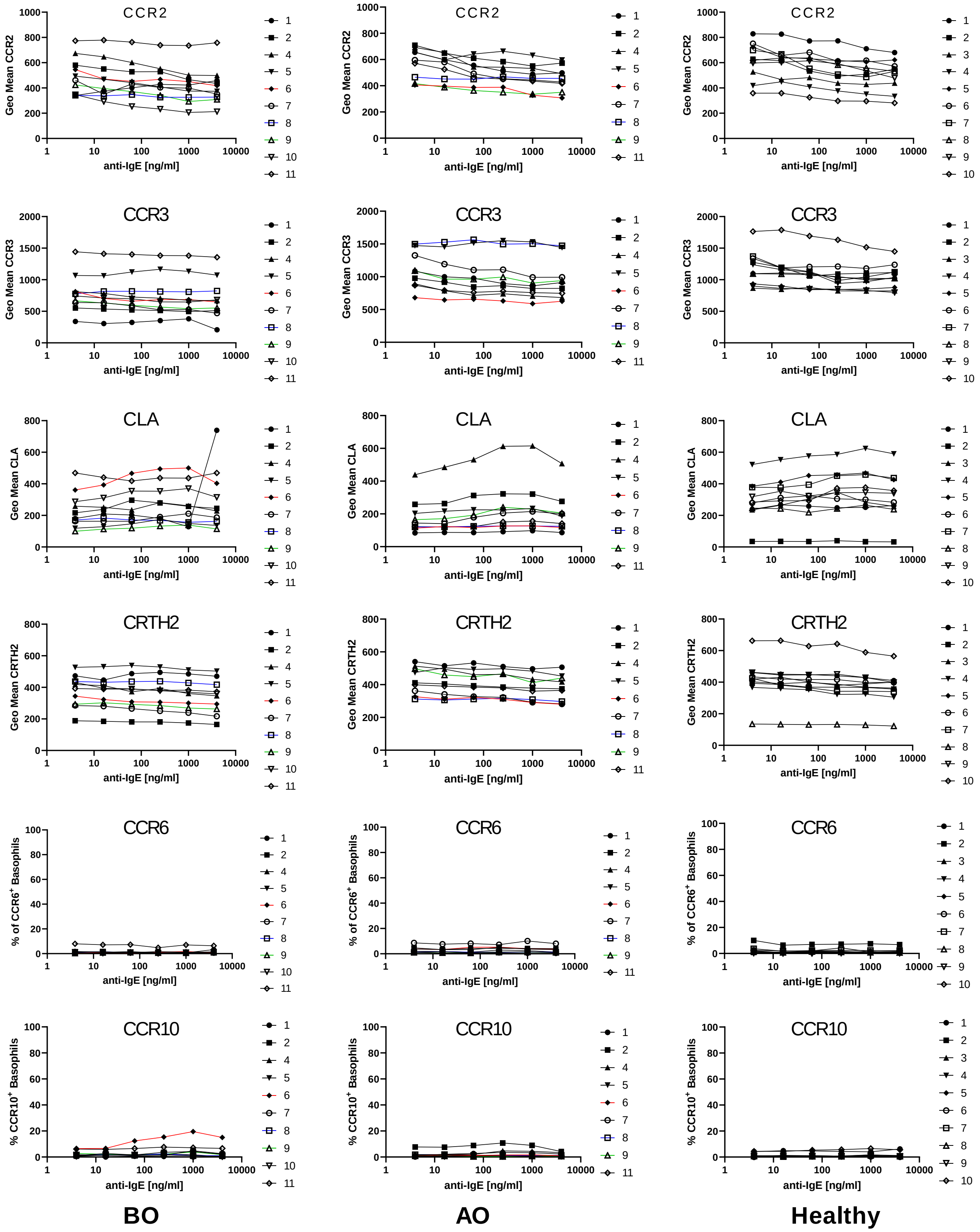

Figure S7, B IgE-mediated stimulation – an expression of CCR2, CCR3, CLA, CRTH2, CCR6, and CCR10

Whole blood basophils were stimulated with serial dilution of anti-IgE (4-4000 ng/mL) – X-axis. Change in the characterized receptors is presented on the Y-axis as GeoMean or % of receptor-positive basophils.

BO and AO refer to patients' groups before and after Omalizumab treatment, respectively; Left, middle, and right panels refer to BO (1-2, 4-11), AO (1-2, 4-9, 11), and healthy (1-10) groups, respectively.

	Resting			*anti-IgE*		
	Healthy	*BO*	*AO*	*Healthy*	*BO*	*AO*
**CCR2**	✓	↓ (red)	↓ (red)	x x	x x	x x
**CCR3**	✓	↓ (red)	↓ (red)	x x	x x	x x
**CLA**	✓	✓	✓	x x	x x	x x
**CRTH2**	✓	✓	✓	x x	x x	x x
**CCR4**	✓	✓	✓	↑ (green) x	↑ (green) x	↑ (green) x
**CCR6**	-	-	-	x x	x x	x x
**CCR8**	✓	↑ (green)	✓	↑↑ (green)	↑ (green) x	↑ (green) x
**CCR10**	-	-	-	x x	x x	x x
**CCX-CKR**	✓	↑ (green)	✓	↑↑ (green)	↑ (green) x	↑ (green) x
**XCR1**	-	-	-	↑ (green) x	↑ (green) x	↑ (green) x
**CD88**	✓	✓	✓	x x	x x	x x
**FPR1**	✓	✓	✓	x x	↑ (orange) x	x x
**CD109**	✓	✓	✓	x x	↓ (orange) x	x x
**IL-31RA**	✓	✓	✓	↓ (red) x	↓ (red) x	↓ (red) x
**PD-L1**	✓	✓	✓	x x	x x	x x
**CD87**	✓	✓	↑ (green)	↑ (green) x	↑ (green) x	↑ (green) x
**CD210**	-	-	-	x x	x x	x x

	fMLP			*C5a*		
	Healthy	*BO*	*AO*	*Healthy*	*BO*	*AO*
**CCR2**	↓ (red) x	x x	x x	x x	x x	↓ (red) x
**CCR3**	x x	x x	x x	x x	x x	x x
**CLA**	x x	x x	x x	x x	x x	x x
**CRTH2**	x x	x x	x x	x x	x x	x x
**CCR4**	↑ (green) x	x x	↑ (green) x	↑ (green) x	x x	↑ (green) x
**CCR6**	x x	x x	x x	x x	x x	x x
**CCR8**	↑ (green) x	↑ (green) x	↑ (green) x	↑ (green) x	x x	↑ (green) x
**CCR10**	x x	x x	x x	x x	x x	x x
**CCX-CKR**	x x	↑ (green) x	x x	x x	x x	x x
**XCR1**	↑ (green) x	↑ (green) x	↑ (green) x	↑ (green) x	↑ (green) x	↑ (green) x
**CD88**	x x	x x	x x	x x	x x	x x
**FPR1**	x x	x x	x x	x x	x x	x x
**CD109**	x x	x x	x x	x x	x x	x x
**IL-31RA**	↓ (red)	↓↓ (red)	x x	x x	x x	x x
**PD-L1**	x x	x x	x x	x x	x x	x x
**CD87**	↑ (green) x	↑ (green) x	x x	x x	x x	x x
**CD210**	x x	x x	x x	x x	x x	x x

Table S1 Overview of receptor expression on basophils from chronic urticaria patients and healthy subjects. Expression of investigated receptors on resting and activated basophils from chronic urticaria patients and healthy individuals.

Resting column – "checkmark" - an expression of a receptor on basophils, "-" – no expression of a receptor on basophils, red/green arrow – reduced/increased expression of a receptor on basophils in chronic urticaria compared to healthy, exception CD87 AO vs. healthy;

Anti-IgE/fMLP/C5a/SP columns – 1st sign refers to the difference between resting vs. stimulated, 2nd sign refers to the difference between BO and healthy, "x" – no change; red/orange/green arrow – reduced/tendency of reduced or increased/ tendency of increased expression of a receptor

BO and AO refer to patients' groups before and after Omalizumab treatment
